# 

**Published:** 1888-07-28

**Authors:** 


### THE STUDENT'S COLUMN.

### ROYAL COLLEGE OF PHYSICIANS AND SURGEONS, EDINBURGH, AND FACULTY OF PHYSICIANS AND SURGEONS, GLASGOW.

### MEDICAL EXAMINATIONS.

The Quarterly Examinations in Edinburgh for the Triple Qualifications took place in July, with the following results:—

#### FIRST EXAMINATION.

Of 49 candidates, the following 35 passed:—Philip Dansey Addis, Bristol; Clifton Sturt, London; Joseph Welsh, Newtownbutler; Henry Taaffe Londonderry; John Cuthbertson Walker, Helensburgh; William Daunt, Dublin; Robert Phillip Cooke, County Roscommon; Wilfred Jameson, Carlow; Edward McCarthy, Kinsale; Daniel John O'Mahony, Cork; George Bridgeford Proctor, Birkenhead; Francis Weatherdon Toms, London; John Timothy Buckley, County Kerry; Alfred Jackson, County Cork; Thomas William Clay, Anglesea; Penry Foster James, Glamorganshire; Arthur Thomas Kimber, India; Horace Allonby Smit, Cape Colony; Charles Howard Roberts, Cape Colony; Frederick John Bonnalie, Chester; Thomas William Edward Waddington, Blackburn; Samuel Haldane Heald, Wakefield; Angus John Macdonald, South Australia; John Joseph Brennan, Kilkenny; Andrew Lees Smith, Hamilton; Thomas Joseph Frost, County Clare; Lionel Selfe Wells, Australia; John Cumming, Banffshire; Philip Graham Findlay, Nairn; John Warburton Wilkinson, Queen's County; Thomas Peter Wells, Blackburn; Maurice Joseph Hickie, Castletownroche; Edward Hughes, Wales; John Francis Butler-Hogan, Dunmanway; and David Samuel Browne, Mullaglass.

#### SECOND EXAMINATION.

Of 59 candidates, the following 33 passed:—Nathaniel Henry Runciman, Cork; John Dunne, County Cork; Edmond William St. Vincent Ryan, Cork; John Francis Roden, Edinburgh; Richard Patrick Byrne, Cork; Gordon Trafforde Tuke, Edinburgh; Simon Ryan, Armagh; Clifton Sturt, London; Albert Edward Wynne, Stafford; Joshua Hamilton Hart, Yorkshire; Robert Love, County Antrim; Miss Beatrice Mary Harrison, Brighton; James Downie Watt, Leamington Spa; Vernon Francis Allen, County Cork; Philip Dansey Addis, Bristol; Richard Fitzgerald, County Cork; Paul Madden Sheedy, County Cork; William Brough Arthur, Dundee; Frederick Charles Foster, Canterbury, New Zealand; Ernest Victor Eames, Donegal; David Illtyd Jones, S. Wales; Arthur Edmund Shepherd, Adelaide; Alexander Vincent Bowen, Preston; James Henry Syme Grant, Dunfermline; Albert Edward William Burns, Chatham; Frederick Albert Capps, Portsmouth; William Johnson Langley, County Waterford; Patrick Francis O'Hagan, Longford; John Francis Butler-Hogan, Dunmanway; Jeremiah Berry, Castletownroche; William Cornelius Rainbury, Queenstown; John Robert Mason, Lancashire; and Percy Stainsby, Yorkshire.

#### FINAL EXAMINATION.

Of 60 candidates, the following 30 passed, and were admitted L.R.C.P.E., L.R.C.S.E. and L.F.P. and S.G.:—Thomas McCubbin, Kirkintilloch; David Scott Moncrieff, Edinburgh; Gilbert Gordon, Ontario; Joseph Adam Nolan, Kilkenny; Thomas Everard, India; Richard Griffith, Carnarvon; John Richard Haigh Dyson, Honley; William Wendt Margenout, Colombo, Ceylon; Charles Barrie Taylor, Manchester; Miss Lilian Agnes Hester Jenkins, Tenby; Rupert Wilberforce Clayton, Wrexham; Lucien Milbourne Clark, Kingston, Jamaica; Nathaniel Henry Runciman, Cork; Robert Trimble, Strabane; William Dwyer Russell, County Tipperary; Thomas Sprot Allan, Glasgow; Alexander Lang Murray, Belfast; Newton John Newbould, Toft; Charles Albert Wickham, County Longford; John Henry Briggs, Yorkshire; Charles Edward Lownds, Newcastle-on-Tyne; Charles Pearce, Santiago, Chili; Andrew Davidson, Bangor, County Down; Alfred Dorriforth Vardon, Calcutta; Marius François Xavier Nallètamby, Mauritius; Edward Treharne, Glamorganshire; Harry Evelyn Mahonie, Sheffield; William Keiller, Midlothian; William Joseph Ryan, Limerick and Walter Halliburton Macdonald, Inverness-shire.

### "The Hospital" Library of New and Standard Works.

For all information respecting the appearance of Titles, Prices, etc., of Books in this page, apply to Mr. W. H. Howe, office of The Hospital, 38, Old Jewry.

### Medical Library.

Abdomen; Diseases of the. By S. O. Habershon. 21/- *Churchill.*
Cancer: Its Allies, and other Tumours. By F. A. Purcell 10/6 *Churchill.*
Diphtheria: Its Nature and Treatment. By Sir Morell Mackenzie. 5/- *Churchill.*
Ear: The Diseases of the. By A. Hartmann. 9/- *Pentland.*
Eye: On Diseases of the. By C. B. Taylor. *Redway.*
Heart: A Practical Treatise on Diseases of the. By W. H. Walshe. 16/- *Smith and Elder.*
Helps to Health. By Hy. C. Burdett. 1/6 *Kegan Paul.*
Help in Sickness. By Hy. C. Burdett. 1/6 *Kegan Paul.*
Liver: Diseases of the. By Geo. Harley. 21/- *Churchill.*
Lungs: A Practical Treatise on Diseases of the. By W. H. Walshe. 16/- *Smith and Elder.*
Midwifery for Midwives. A Manual of. By Fancourt Barnes. 6/- *Smith and Elder.*
Nervous System; A Manual of Diseases of the. By W. R. Gowers. 12/6 *Churchill.*
Poisons: their Effects and Detection 16/- By A. W. Blyth. 16/- *Griffin and Co.*
Ringworm: Its Diagnosis and Treatment. By A. Smith. *Lewis.*
Skin: Diseases of the. By H. Radcliffe Crocker. 21/- *H. K. Lewis.*
Skin Diseases. By Malcolm Morris. 7/6. *Smith and Elder.*
Throat: Diseases of the Mouth, Throat, and Nose. By P. Schech. 9/- *Pentland.*

### Library of Biography and History.

Bacon. By R. W. Church (Dean of St. Paul's). 5/- *Macmillan.*

Gladstone; Anecdotes of. By an Oxford Man. 1/- *Hamilton.*

Greece: A History of. Part I. By Edwin Abbott. 10/6. *Rivingtons.*

Henry II. By Mrs J. R. Green. 2/6. *Macmillan.*

Mitchell: Life of John. By Wm. Dillon. 2 vols. 21/- *Kegan Paul.*

### Library of Christian Literature.

A Century of Christian Progress and its Lessons. By J. Johnston. 3/- *Nisbet*

Faithful Departed; The Present State of the. By Rev. Geo. Body. 1/- *Masters.*

St. John, the Author of the Fourth Gospel. By H. H. Evans. 6/- *Nisbet.*

The Victory of the Cross. By B. F. Westcott. 3/6 *Macmillan.*

### Dictionaries and Encyclopædias.

Everybody's Pocket Cyclopædia. By E. Sheldon. 6d. *Saxon and Co.*
Middle English: A Concise Dictionary of. By A. L. Mayhew—W. W. Skeat. 7/6 *Froude.*
National Biography; Dictionary of. By Leslie Stephens 15/- *Smith and Elder.*
New English Dictionary on Historical Principles. Edited by James Murray. Part 4 (Bra-Cass). 12/6 *Froude.*

### Library of Fiction.

A Bitter Repentance. By Lady Virginia Sandars. 3 vols. *Hurst and Blackett.*

A Woman's Face. By F. Warden. 3 vols. *Ward and Downey.*

Children of Gibeon. By Walter Besant. 2/- *Chatto and Windus.*

Eve. A Romance. By Author of "John Herring." 2 vols. *Chatto and Windus.*

Jess. By H. Rider Haggard 2/6 *Smith and Elder.*

Our Saturday Nights. By James Greenwood. 1/- *Diprose.*

St. Bernards: The Romance of a Medical Student. By Æsculapius Scalpel. 2/- *Sonnenschein.*

The Elect Lady. By Geo. Macdonald. 1/- *Kegan Paul.*

### CHANGE OF OFFICE AND PUBLISHER.

All business communications should be addressed to the Manager, Office of The Hospital, 38, Old Jewry (Cheapside end). All Cheques and Post Office Orders should be made payable to J. H. S. Hanning, Secretary, 38, Old Jewry, E.C., crossed Lloyds, Barnetts, and Bosanquet's Bank (Limited).

### To Secretaries and Matrons.

The Editor will be glad to have a copy of the Regulations, and also of every Report issued from the Press of Asylums, Hospitals, Convalescent and Nursing Institutions, as well as those of other Charities, so soon as they are published, and an early copy in duplicate of all appeals for funds. Notices of Annual and other Meetings, Festival Dinners, etc., will be welcomed. All such communications should be addressed to *The Editor, The Lodge, Porchester Square, London, W.* Any superintendent, secretary, matron, or other chief official who will regularly send such documents and information will be registered to receive free of cost a cover for binding The Hospital in half-yearly volumes on sending name and address to the publisher.

### AMUSEMENTS WITH PROFIT FOR ALL AGES.

**Rules.**—All answers must be addressed to the PRIZE EDITOR, THE HOSPITAL, 38, Old Jewry, Cheapside, London, E.C., not later than the *first* post on Thursday next. The full name and address must in all cases be given, but a *nom de plume* may be added for publication. Only one side of the paper must be written on.

### FOR MEN AND WOMEN.

### First Quarterly Poetical Competition.

Commenced May 26th, 1888; ends August 25th, 1888.

This quarter a PRIZE of ONE GUINEA will be given to the competitor who gains the highest number of marks for original poems, on any subjects suitable for insertion in this paper. All contributions sent in will be credited according to merit, eighteen marks being the highest score for any one contribution. This competition to alternate weekly with the set acrostic competition.

### Acrostic Competition.

This quarter the original acrostic competition will be discontinued, and A PRIZE of HALF-A-GUINEA will be given to the competitor who gains the highest score for solution of acrostics set. *One mark* only will be given to the competitors who solve all the lights of each acrostic.

Exception will be made in the case of *quotation acrostics*, when *two marks* will be given, *one* for the correct solution of all *lights*, and *one* mark for the *source* of the quotations.

This week credit will be given for

### No. 5.—Original Poem.

### Results of No. 4 Original Poem.

Dodo (15), S. M W. (14), A. M. E. (13), Patience (13), K. M. (13), Hesperornis (11).

### The Old Mill Stream.

DOWN by the mill, where the shadows lie
Soft and cool 'neath the sunniest sky,
Couched on a bank of the brightest green
Pied with the buttercups' golden sheen,
Dreaming list, as the shadows steal,
To the noisy whirr of the old mill-wheel.

In fancy I roam o'er the upland fell
To the streamlet's rise, where the heatherbell
Nods and quivers, and swings at ease
To the playful touch of the mountain breeze;
And the water trickles 'mid mosses cool
In silver threads to the hill-top pool.

Ruggedly cradled they pause in rest,
Calm as the babe's on a mother's breast;
Gathering strength for the work of life
In nook unbroken by sound of strife,
Reflecting the clouds and the sunlit skies,
As the mother's face in her baby's eyes.

But rugged or loving, what clasp can stay
The stern soft touch of the Lord of Day,
As He gathers his own to regions fair
The babe to heaven, the drops to air?
Where, waiting in beauty, they strength renew
For the work He has bidden them each pursue.

Floating above us in forms so fair
Veil they the fields from the noontide glare,
Till, at His bidding, they swift speed forth
Eastward or westward, to south or north,
Pure from His presence to gather and fall,
Where the thirsting clods to their Maker call.

Down thro' the cracks of the parchèd earth
They wind to the spot where the fount has birth,
Then bubble and drip, till with gurgle and splash
They merrily dance 'neath the mountain ash,
Or hurry and scurry by boulder and scaur
To leap down the fall with a noisy roar.

Here in the basin they eddy and swirl,
In fanciful mazes madly whirl,
Sprinkle with spray as the foam they churn,
Trailing ivy and hart's-tongue fern;
Then ripple and wind down the pebbly reach
With the sound of waves on a shingle beach.

On thro' the pasture the streamlet glides
Where meadow-sweet crowns its verdant sides,
Or willows and osiers, reeds and sedge
Cluster in groups at the water's edge,
And lilies deep-anchored brightly gleam
On the placid breast of the silent stream.

Over the dam of the moss-grown weir
Flash in the sunlight the waters clear;
Or under the branches in shadowy cool
Flow on their way to the miller's pool.
While minnow and grayling, roach and bream
Claim tenant right in the old mill stream.

In the deep still pool for awhile they bask,
Waiting the call to their destined task,
Watching the swallows that lightly skim,
Flutter, and wheel at the waters' rim.
With shimmer and shiver 'mid glint and gleam
The zephyr is wooing the old mill stream.

Short time they tarry, the work to be done
Calls them away from the breeze and the sun;
Over the shoot for a dash to the wheel
Slowly but surely the waters steal;
Then battle in gloom to the foaming race,
And hurry along to their destined place.

DODO.

### THE WORD COMPETITION.

Third Quarterly Competition commenced May 5th, 1888; ends August 4th, 1888.

Three prizes of one guinea, 15*s.* and 10*s.* 6*d.* will be given each quarter to the three competitors who obtain the highest number of marks by making the largest number of words derived from the word or phrase set for anatomical dissection; that for this, the *twelfth* week of the quarter, being

"LUCERNE."

OBSERVE.—Proper names, abbreviations, foreign words, words of less than four letters, and repetitions are barred; plurals, and past and present participles of verbs are allowed. Nuttall's *Standard* dictionary only to be used.

N.B.—All words sent in must be arranged *alphabetically*, with correct total affixed.

### Results of Tenth Week Puzzles.

*Pension.*—Alicia (30), Patience (30), Lightowlers (30), Lauriston (30), Reldas (29), Dodo (28), Puff (27), Sym (26).

### Notices to Correspondents.

*W. G. Roffey.*—The Acrostic Competition is discontinued.

### THE CHILDREN'S CORNER.

PRIZES.

FOR MOST CORRECT ANSWERS TO PUZZLES.

This Commenced October 1st, 1887.

One Pound a Month.

A PRIZE OF THREE GUINEAS

to the Competitor who shall have sent in the largest number of correct or best answers during the twelve months.

### Puzzles.—Third Week.

No. 9. The divided square. Divide the number 13 into three parts so that their squares may have equal differences, and the sum of their squares may be 75.

No. 13. DOUBLE ACROSTIC.—*The Globe divided.*

1. Wisdom blended with the earth
Issues but in folly's birth.

2. Backwards, forwards, all the same,
I am but a lady's name.

3. Tho' of brittle comformation.
Falling is my occupation.

4. Patient worker though I be,
Still the rebel lives with me.

5. Among the sheaves of corn she stood,
The type of faithful womanhood.

No. 11. ENIGMA.

I am, dear children—let me see—
I am a flower, a shrub, a tree;
A bird, a beast, a man, a woman—
Is this relation strange, uncommon?
All this is singular I own
But am I singular alone?
So or not so, I claim, you'll see,
The attribute *plurality*.

No. 12. SQUARE WORD.—1. A resting place. 2. A girl's name. 3 Charity. 4. A lesson.

. . . .<br>. . . .<br>. . . .<br>. . . .

### Results of Second Week Puzzles.

No. 5. C-o-m-i-c=Comic.

No. 6.

G<br>A L E<br>P L A I T<br>G L A S G O W<br>P A G E T<br>C O W<br>W

No. 7. New-South Wales=New South Wales.

No. 8.

S underlan D<br>Y okoham A<br>R ochefor T<br>I rvin E<br>A mien S

### Marks

| | July 14th. | | Total of First and Second Weeks. |
|---|---|---|---|
| A C. K. | 4 | .... | 7 |
| Alsie | 3 | .... | 5 |
| Boadicea | 4 | .... | 7 |
| Curly | 4 | .... | 7 |
| Geranium | 4 | .... | 6 |
| Happy Eliza | 4 | .... | 4 |
| Hercules | 4 | .... | 6 |
| Jack | 4 | .... | 7 |
| Poodle | 2 | .... | 5 |
| Thanet | 4 | .... | 7 |
| Scrooge | 4 | .... | 8 |
| Young Surgeon | 4 | .... | 6 |
| Gem | — | .... | 4 |

**Notice to all Correspondents.**—N.B.—All letters referring to this page, which do not arrive at 38, Old Jewry, Cheapside, London, E.C., by the *first post on Thursdays*, and are not addressed PRIZE EDITOR, will in future be disqualified and disregarded.

No one will be permitted to win the Monthly Prizes twice in any one year, the Annual Prize being offered especially to prevent this.

